# Tempol Ameliorates and Prevents Mechanical Hyperalgesia in a Rat Model of Chemotherapy-Induced Neuropathic Pain

**DOI:** 10.3389/fphar.2016.00532

**Published:** 2017-01-16

**Authors:** Hee Kee Kim, Seon-Hee Hwang, Salahadin Abdi

**Affiliations:** Department of Pain Medicine, Division of Anesthesiology and Critical Care, The University of Texas MD Anderson Cancer CenterHouston, TX, USA

**Keywords:** chemotherapy, neuropathic pain, free radical, inflammatory cytokines, paclitaxel, tempol

## Abstract

Chemotherapy-induced neuropathic pain is difficult to treat and prevent. Tempol decreases cellular superoxide radical levels and oxidative stress. The aims of our study were to investigate the analgesic and preventive effects of tempol on paclitaxel-induced neuropathic pain in rats and to identify the associated mechanisms of action. Neuropathic pain was induced with intraperitoneally injected paclitaxel on four alternate days in male Sprague–Dawley rats. Tempol was administered systemically as a single injection and a continuous infusion before or after the injection of paclitaxel. The mechanical threshold for allodynia, protein levels, and free radical levels were measured using von Frey filaments, Western blotting, and live cell imaging, respectively. After the rats developed neuropathic pain behavior, a single intraperitoneal injection and continuous infusion of tempol ameliorated paclitaxel-induced mechanical allodynia. Systemic infusion of tempol in the early phase of the development of pain behavior prevented the development of paclitaxel-induced pain behavior. Paclitaxel increased the levels of phosphorylated protein kinase C, phosphorylated nuclear factor κB, phosphodiesterase 4D (PDE4D), IL-1β, and monocyte chemoattractant protein-1 in the lumbar dorsal root ganglia; however, tempol decreased these levels. Paclitaxel also increased superoxide levels in a culture of primary dorsal root ganglion cells and tempol decreased these levels. In conclusion, tempol alleviates and prevents chemotherapy-induced neuropathic pain in rats by reducing the levels of inflammatory cytokines and free radicals in dorsal root ganglia.

## Introduction

Chemotherapeutic agents, including taxanes (e.g., paclitaxel, docetaxel), vinca alkaloids (e.g., vincristine, vinblastine), platinum agents (e.g., cisplatin, carboplatin, oxaliplatin), and others (e.g., thalidomide, bortezomib, lenalidomide), produce peripheral pain in the distal extremities in a symmetrical glove and stocking distribution (Yazdani and Abdi, 2014). Chemotherapy-induced neuropathic pain is a dose-limiting adverse effect that can take place at any time during the course of the treatment or even after its termination (Windebank and Grisold, 2008). This neuropathy can significantly decrease the overall quality of life in cancer patients (Mols et al., 2014; Rivera and Cianfrocca, 2015).

To date, analgesic drugs such as opioids, non-steroidal anti-inflammatory agents, anticonvulsants, antidepressants, and sodium channel blockers show little or no analgesic effects in paclitaxel-induced neuropathic pain (PINP) models (Xiao et al., 2008). It has also been reported that glutamine, glutathione, *N*-acetylcysteine, oxcarbazepine, and xaliproden did not prevent chemotherapy-induced neuropathy (Wolf et al., 2008). On the other hand, there have been several preclinical reports indicating cannabinoids might have some effects in reducing chemotherapy-induced peripheral neuropathy (Deng et al., 2012, 2015; Khasabova et al., 2012; Guindon et al., 2013; Vera et al., 2013; Harris et al., 2016), but their clinical efficacy has not been proved yet. Therefore, currently, no effective medications are available to treat or prevent neuropathic pain (Lee and Swain, 2006; Wolf et al., 2008; Hershman et al., 2014) and new alternatives need to be explored.

Several pain models have shown that systemic or spinal administration of free radical scavengers, including phenyl *N*-tert-butylnitrone and vitamin E, reduce pain behavior (Kim et al., 2004, 2006). In addition, tempol, a membrane-permeable superoxide dismutase mimetic, was reported to attenuate zymosan-induced visceral pain behavior and peroxynitrite-enhanced carrageenan-induced hyperalgesia (Khattab, 2006; Ji et al., 2015). Tempol is a member of nitroxide compounds and reacts with superoxide anion to form hydrogen peroxide. In addition, the sensitivity of tempol was greatest for hydroxyl radical, intermediate for hydrogen peroxide, and least for superoxide radical (Soule et al., 2007; Wilcox and Pearlman, 2008). The toxic doses of tempol are about 172.25–344.5 mg/kg when animals were intraperitoneally injected (Hahn et al., 1998). Tempol reduced oxidative damage in cerebral synaptosomes from gerbils and reduced exacerbated hypoxic brain damage (Howard et al., 1996; MacGregor et al., 2003). Tempol blocked the enhanced *N*-methyl-D-aspartate-induced neurotoxicity in cultured cortical brain cells (Hewett et al., 1994; Teichner et al., 2003). In addition, several studies have reported that tempol reduced tumor growth and incidence of cancer. Tempol decreased glioma formation in rats and delayed the development of tumors, and prolonged the lifespan of cancer-prone mice (Gariboldi et al., 2003; Erker et al., 2005; Zhang et al., 2008). However, the analgesic and preventive effects of tempol on chemotherapy-induced neuropathic pain have not been studied. The aims of this study were to investigate (1) the analgesic effects of tempol on PINP in rats, (2) the preventive effects of tempol on the development of PINP in rats, and (3) the mechanisms of action of tempol in the dorsal root ganglia (DRGs).

## Materials and Methods

### Experimental Animals

Male adult Sprague–Dawley rats (200–350 g; Harlan Sprague–Dawley Company, Houston, TX, USA) were used for the experiment. They had free access to food and water and were housed in a room with a normal light-dark cycle (light cycle: 7:00 a.m. to 7:00 p.m.). All animals were habituated for 1 week before the experiments. The experimental protocol was approved by the institutional animal care and use committees of The University of Texas MD Anderson Cancer Center.

### Paclitaxel-Induced Neuropathic Pain

Paclitaxel (Sigma, St. Louis, MO, USA) was dissolved in a vehicle solution (4% dimethyl sulfoxide and 4% Tween 80 in sterile saline) and was injected intraperitoneally at a dose of 2 mg/kg on days 0, 2, 4, and 6 (Polomano et al., 2001; Kim et al., 2010). Control rats were injected with the same volume of vehicle without paclitaxel.

### Measurement of Mechanical Allodynia

To measure mechanical allodynia, we used a behavior test that has been described previously (Chaplan et al., 1994). Briefly, rats were placed in a plastic chamber on top of a mesh screen, and the mechanical threshold of the left hind paw was determined by the up-down method (Dixon, 1980) using monofilaments (0.45–14.45 g). A filament was applied to the most sensitive parts of the paw’s plantar surface – the center of the paw or the base of the third or fourth toes – for 3–4 s. A sudden withdrawal of the foot during stimulation or immediately after removal of the filament was considered to be a positive response. A 50% mechanical threshold value was calculated. The investigator who conducted the behavioral tests was blinded to the control or treatment status of the rats.

### Western Blot Analysis

The rats were anesthetized deeply with 4% isoflurane for induction for 5 min and then 3% for the maintenance. During anesthesia, rats were removed hair, opened chest cavity, and then perfused with cold saline. The L1-6 DRGs were removed, frozen in liquid nitrogen, and then stored in deep freezer (-80°C). They were homogenized in 150 μl of RIPA cell lysis buffer with a protease inhibitor on ice by homogenizer four times for 20 s (interval time: 2 min) and centrifuged at 17,000 × *g* at 4°C for 10 min. And then supernatants were loaded in 10% sodium dodecyl sulfate-polyacrylamide gels and transferred to polyvinylidene fluoride membranes. Blots were incubated with primary antibody against IL-1β (1:1000; Santa Cruz Biotechnology, Dallas, TX, USA), MCP-1 (1:500; Santa Cruz Biotechnology, Dallas, TX, USA), PDE4D (1:5000; Abcam, Cambridge, MA, USA), phosphorylated nuclear factor κB (p-NF-κB, 1:1000; Cell Signaling Technology, Danvers, MA, USA), phosphorylated protein kinase C (p-PKC, 1:1000; Cell Signaling Technology, Danvers, MA, USA), and GAPDH (1:5000; Santa Cruz Biotechnology, Dallas, TX, USA) overnight at 4°C. The blots were then incubated with anti-rabbit horseradish peroxidase-conjugated secondary antibody (1:5000; GenDepot, Katy, TX, USA) or anti-goat horseradish peroxidase-conjugated secondary antibody (1:5000; GenDepot, Katy, TX, USA). The immunoblots were analyzed with a chemiluminescence detection system and normalized to GAPDH. For equalizing protein loading, GAPDH expression was used as a control.

### Behavioral Testing for Sedation

Behavioral testing for sedation was based on five-point scales of posture (0 = normal, 4 = flaccid atonia) and righting reflexes (0 = struggles, 4 = no movement; Devor and Zalkind, 2001; Kim et al., 2004). Sedation was assessed immediately after each pain behavior test.

### Administration of Tempol

**FIGURE 1:**
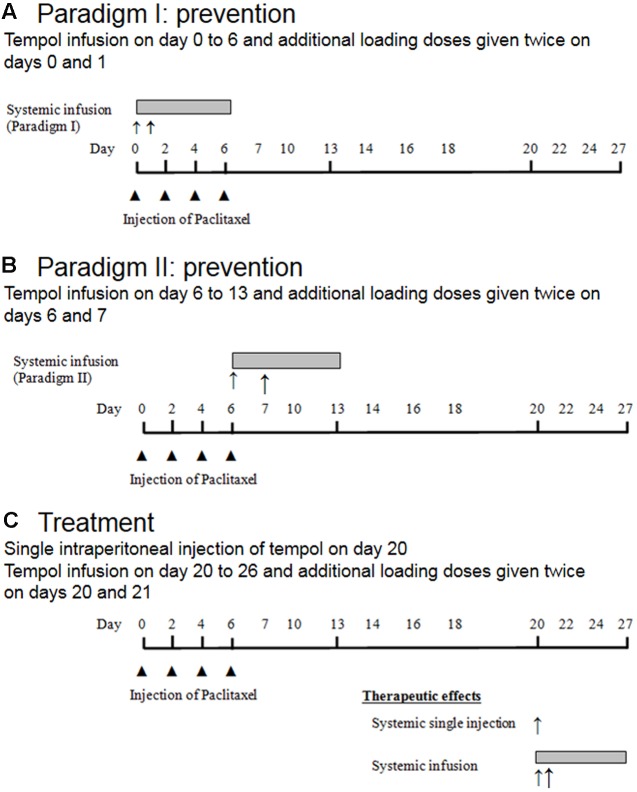
**Schematic overview of the experimental design**. Note, 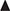 represented single intraperitoneal injection of paclitaxel, ↑ represented single intraperitoneal injection of tempol, and 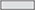 represented intraperitoneal infusion of tempol.

This study consisted of two parts: (1) Assessment of the therapeutic effects of tempol on PINP, given as a single intraperitoneal injection (50, 100, or 200 mg/kg) or as intraperitoneal infusion (10 mg/day for 7 days), and (2) investigation of the preventive effects of tempol given as intraperitoneal infusion on development of PINP (**Figures 1A–C**).

#### Assessment of the Therapeutic Effects of Tempol

For administration of tempol as a single intraperitoneal injection, on day 20 after the first paclitaxel injection, 24 rats were divided into four groups. After the rats developed neuropathic pain behavior, they were randomly assigned to one of three treatment groups or a control group. Rats in the treatment groups (six rats per group) received 50, 100, or 200 mg/kg tempol in 5 mL/kg saline, and rats in the control group received a single 5-mL/kg injection of saline (**Figure 1C**).

For administration of tempol as a systemic intraperitoneal infusion (**Figure 1C**), on the 20th day after the first paclitaxel injection, the rats that developed neuropathic pain behavior were randomly assigned to either a treatment group (tempol, six rats) or a control group (vehicle, five rats) before the insertion of a mini-osmotic pump (Alzet model 2001; Alzet, Cupertino, CA, USA). The rats in the treatment group received an infusion of tempol at a rate of 1 μL/h for 7 days (10 mg/day). Additionally, loading doses of tempol (200 mg/kg) were administered on days 20 and 21 after the first paclitaxel injection. The rats in the control group received equivalent volumes of saline via the pump and as loading doses (5 mL/kg).

#### Assessment of the Preventive Effects of Tempol

Tempol (10 mg/day) was infused intraperitoneally (Alzet model 2001 mini-osmotic pump) by one of the following methods of administration. In paradigm I, a bolus of tempol was injected intraperitoneally at a dose of 200 mg/kg on days 0 and 1 and continuously infused intraperitoneally for 7 days (days 0 through 6; **Figure 1A**). In paradigm II, the same dose of tempol was given as a bolus on days 6 and 7, and continuous infusion was administered on days 6 through 13 (**Figure 1B**). Mechanical allodynia was measured on days 0, 2, 4, 6, 7, 9, 10, 11, 12, 13, 14, 16, 17, 18, 19, 20, 24, 26, 30, 34, 37, and 40. We measured the animal’s body weight regularly after the aforementioned behavioral tests. There was normal increase in their body weight. Furthermore, while observing animals, we did not notice any stress.

### Intraperitoneal Implantation of the Mini-Osmotic Pump

The mini-osmotic pump was implanted intraperitoneally according to the manufacturer’s protocol. Briefly, a midline skin and peritoneal wall incision was made in the lower abdomen of rats that had received isoflurane anesthesia in oxygen. The pump was filled with tempol or saline and inserted into the peritoneal cavity. The musculoperitoneal layer was sutured with silk sutures, and the skin incision was closed with wound clips. Anesthesia was discontinued, and the animals were allowed to recover from anesthesia.

### DRG Cell Culture

Dorsal root ganglia cells were cultured as previously described with slight modifications (Shetty et al., 2015). Briefly, under general anesthesia with isoflurane, the lumbar DRGs from L1 to L6 were removed in sterile conditions. The DRGs were then dissociated with 1.25 mg/mL collagenase (Sigma) twice for 1 h at 37°C and mechanical dissociation. Cells were plated on a 35-mm dish (Costar, Corning, NY, USA) coated with poly-*L*-lysine and laminin in Dulbecco’s Modified Eagle’s Medium (DMEM) with 10% fetal bovine serum (FBS), 1% penicillin/streptomycin, and 4 mM glutamine for 5 days in CO_2_ incubator (5% CO_2_, 37°C). DRG cells were plated in a chambered coverglass with cover No. 1.5 borosilicate sterile (four well, Thermo Fisher Scientific, Waltham, MA, USA) in a volume of 1 mL of DMEM with 5% FBS for live cell microscopy studies. The cells were stained with trypan blue, and viability was measured by a Luna automated cell counter (Logos Biosystems, Annandale, VA, USA).

### Live Cell Imaging for Mitochondrial Superoxide Level

Dorsal root ganglia cells were plated in a chambered coverglass with cover No. 1.5 borosilicate sterile (four well, Thermo Fisher Scientific) in a volume of 1 mL of DMEM with 5% FBS. After 24 h incubation, cells were stained with 5 μM MitoSOX (Molecular Probes, Eugene, OR, USA) for 10 min, washed with DMEM twice, and then treated for 2 h with 1 μM paclitaxel in vehicle (0.025% dimethyl sulfoxide in DMEM). MitoSOX is a novel fluorogenic dye and produces red fluorescence by superoxide from only mitochondria in live cells. In addition, MitoSOX can permeate live cells where it selectively targets mitochondria. Red fluorescent intensity in living cells were measured using a Zeiss LSM510 Meta laser scanning confocal system (Carl Zeiss, Inc, Thornwood, NY, USA) in a box incubator (5% CO_2_, 37°C, Reinach, Switzerland). Confocal scanning setting was used at the lowest laser intensity to capture pixels in the range of 0–255. The images were quantified using Volocity imaging software (Improvision, Perkin Elmer, Waltham, MA, USA). The fluorescent threshold was set at 50–255. Data represent the sum of pixels in an image by the area that produced red fluorescence.

### Statistical Analyses

Data were summarized as means with standard errors of the means for the behavioral testing, Western blotting, and live cell imaging. The data were analyzed using the GraphPad Prism 6 and two-way repeated-measures analyses of variance with one repeated factor (time), followed by the Tukey *post hoc* test for behavioral testing and the Mann–Whitney *U* test for Western blotting and cell imaging. In all cases, *P* < 0.05 was considered statistically significant.

## Results

### Tempol Did Not Produce Sedation

All rats treated with single (50, 100, 200 mg/kg) or infusion (10 mg/day) of tempol or vehicle had a score of 0 on both the posture and righting reflex scales, indicating that tempol did not produce sedation. Reviewer recommended to use positive control such as diazepam to validate the sedation experiment. At this time, we did not use positive control because we had reported several publications using this method (Devor and Zalkind, 2001; Kim et al., 2004, 2006, 2010, 2015). In addition, all rats treated with tempol without paclitaxel showed normal behavior including no sedation, no pain behavior, and normal increase in body weight.

### Tempol Increased the Mechanical Threshold of PINP

**FIGURE 2:**
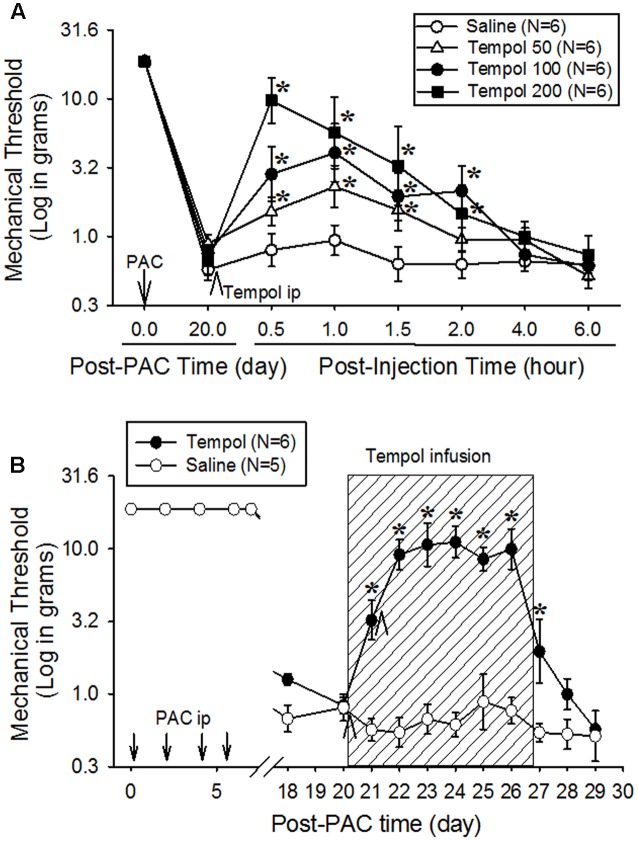
**Analgesic effects of a single systemic injection (A)** or systemic infusion **(B)** of tempol on paclitaxel-induced neuropathic pain (PINP) in rats. Paclitaxel (PAC, 2 mg/kg) was injected intraperitoneally on four alternate days (days 0, 2, 4, and 6), and the mechanical threshold was measured. **(A)** On day 20 after the first paclitaxel injection, 24 rats were divided into four groups, which received an intraperitoneal injection of saline or 50, 100, or 200 mg/kg of tempol (5 mL/kg). Note that the mechanical threshold returned to more than 10 g of mechanical threshold (no pain condition in rats) at 0.5 h after the injection of 200 mg/kg of tempol. **(B)** On day 20 after the first paclitaxel injection, 11 rats were divided into two groups. In the tempol group (*N* = 6), the rats received an intraperitoneal infusion of tempol (10 mg/day) for 7 days (hatched box) in combination with intraperitoneal injections of tempol (200 mg/kg) on days 20 and 21 (arrowheads). In the saline group (*N* = 5), the rats received saline (vehicle) instead of tempol. The systemic infusion of tempol significantly increased the mechanical threshold on day 21, and the threshold remained significantly higher than the threshold in the control group for 8 days. The data are expressed as means with standard errors of the means. The asterisks indicate values that are significantly different (*P* < 0.05) from the corresponding values for the saline group as determined by a two-way repeated-measures analysis of variance with one repeated factor (time) followed by the Tukey *post hoc* test.

Single intraperitoneal injection of tempol at doses of 50, 100, and 200 mg/kg on day 20 increased mechanical threshold in a dose-dependent manner in rats (**Figure 2A**). Moreover, the 200-mg dose significantly increased mechanical threshold from 0.9 to 9.7 g at 0.5 h after injection compared with the saline group, but the threshold returned to baseline at 2 h. We selected 50, 100, and 200 mg/kg based on our preliminary data.

To investigate the extended analgesic effects of tempol, 200 mg/kg was intraperitoneally injected on days 20 and 21, and 10 mg/day was intraperitoneally infused for 7 days (**Figure 2B**). The control group received intraperitoneal injections and infusion of saline. Mechanical threshold was measured each morning. Tempol significantly increased the mechanical threshold over that of the saline group on day 21, and the threshold remained significantly higher on day 27. These data indicate that infusion of tempol produced a prolonged analgesic effect without sedation.

### Tempol Prevented the Development of PINP

**FIGURE 3:**
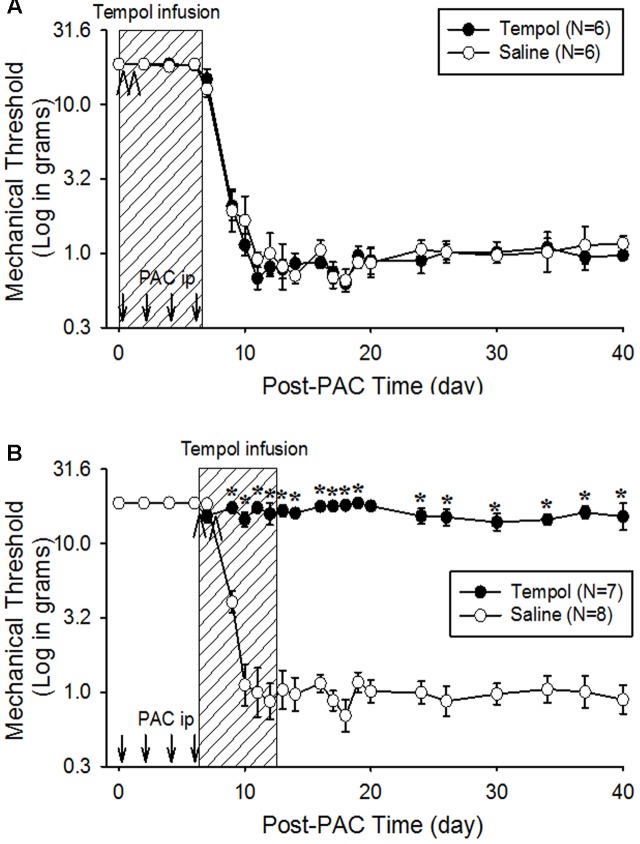
**Preventive effects of tempol on PINP in rats**. Paclitaxel (PAC, 2 mg/kg) was injected intraperitoneally on four alternate days (days 0, 2, 4, and 6), and the mechanical threshold was measured. **(A)** On the day of the first paclitaxel injection, 12 rats were divided into two groups. The rats in the tempol group (*N* = 6), received an intraperitoneal infusion of tempol (10 mg/day) for 7 days (hatched box) and an intraperitoneal injection of tempol (200 mg/kg) on days 0 and 1 (arrowheads) of the infusion period. The rats in the saline group (*N* = 6) received equivalent amounts of saline. The systemic infusion of tempol did not affect the development of PINP. **(B)** On the day of the last paclitaxel injection, another 15 rats were divided into two groups. The rats in the tempol group (*N* = 7) received an intraperitoneal infusion of tempol (10 mg/day) for 7 days (hatched box) and intraperitoneal injections of tempol (200 mg/kg) on days 6 and 7 (arrowheads). The rats in the saline group (*N* = 8) received equivalent volume of saline. The systemic infusion of tempol significantly prevented the development of PINP. The data represent means with standard errors of the means. The asterisks indicate values that are significantly different (*P* < 0.05) from the corresponding values for the saline group as determined by a two-way repeated-measures analysis of variance with one repeated factor (time) followed by the Tukey *post hoc* test.

To examine its preventive effects, tempol (10 mg/day) was infused intraperitoneally for 7 days beginning 1 h before the first paclitaxel injection (day 0) in paradigm I or beginning on day 6 in paradigm II. The early treatment (starting on day 0) did not affect the development of pain behavior (**Figure 3A**). In contrast, the treatment beginning on day 6 completely prevented further development of pain behavior for 40 days in paradigm II (**Figure 3B**). These data indicate that tempol prevented the development of PINP when given during the development phase of mechanical hyperalgesia.

### Paclitaxel Increased the Levels of p-PKC, p-NF-κB, PDE4D, IL-1β, and MCP-1 in DRGs, and Tempol Subsequently Decreased the Levels

**FIGURE 4:**
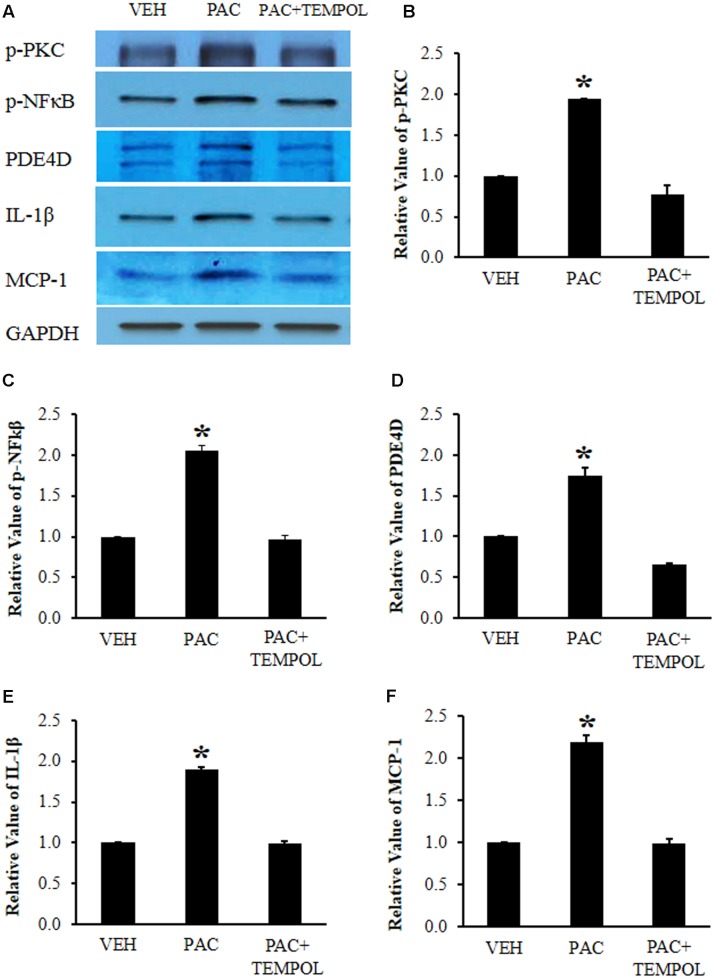
**Paclitaxel increased the levels of phosphorylated protein kinase C (p-PKC), phosphorylated nuclear factor κB (p-NF-κB), PDE4D, IL-1β, and monocyte chemoattractant protein-1 (MCP-1) in rat dorsal root ganglia (DRGs)**. **(A)** Western blot showing the expression of p-PKC, p-NF-κB, PDE4D, IL-1β, and MCP-1 in DRGs after an injection of vehicle (VEH) or paclitaxel (PAC) on day 20. Tempol was infused for 7 days (days 14–20). **(B–F)** Quantification of p-PKC, p-NF-κB, PDE4D, IL-1β, and MCP-1 in DRGs. Note that paclitaxel increased the levels of p-PKC, p-NF-κB, PDE4D, IL-1β, and MCP-1 in rat DRGs, and tempol subsequently decreased these protein levels. The data are expressed as means ± standard deviations for three rats. The asterisks indicate values that are significantly different (*P* < 0.05) from the values for the vehicle group as determined by the Mann–Whitney *U* test.

To examine the levels of signaling molecules, paclitaxel (2 mg/kg on days 0, 2, 4, and 6) or vehicle (4% dimethyl sulfoxide and 4% Tween 80 in saline) was intraperitoneally injected, and L1-6 DRGs were collected on day 20 after the first injection of paclitaxel or vehicle for Western blot analysis. Paclitaxel significantly increased the levels of p-PKC (1.9 times), p-NF-κB (2.1 times), PDE4D (1.7 times), IL-1β (1.9 times), and MCP-1 (2.2 times) in the DRGs compared to those in the vehicle control group (**Figures 4A–F**). Subsequently, tempol was infused for 7 days (days 14–20), and lumbar DRGs were removed on day 20 for Western blot analysis. Tempol decreased the levels of p-PKC, p-NF-κB, PDE4D, IL-1β, and MCP-1 in the DRGs of paclitaxel-treated rats to the levels in the DRGs of vehicle-injected rats (**Figures 4A–F**).

### Paclitaxel Increased Mitochondrial Superoxide Levels in DRG Cell Culture, and Tempol Subsequently Decreased the Levels

**FIGURE 5:**
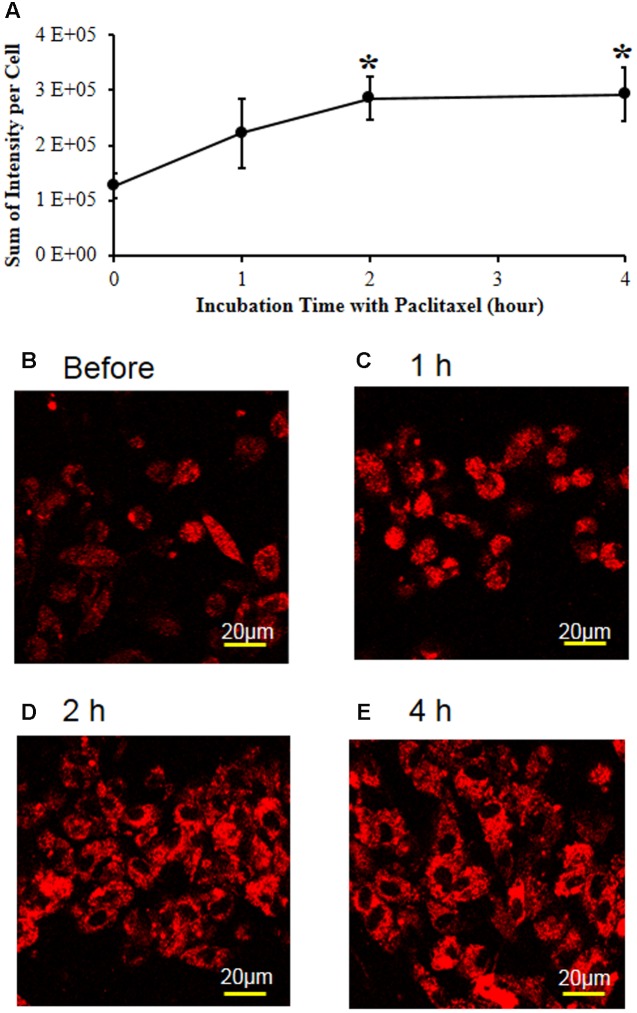
**Paclitaxel increased mitochondrial superoxide levels in the primary DRG cell culture**. **(A)** The L1-6 DRGs were isolated from an adult male Sprague–Dawley rat. The isolated DRG cells were plated in a chambered coverglass in DMEM media for live cell imaging. Note that paclitaxel (1 μM) significantly increased the MitoSOX-detected red fluorescence intensity in the primary DRG cell cultures. The data are expressed as means ± standard deviations for three independent experiments. The asterisks indicate values that are significantly different (*P* < 0.05) from the values before treatment with paclitaxel as determined by the unpaired *t*-test. **(B–E)** The MitoSOX-detected red fluorescence intensity was measured in live cell imaging before **(B)** and 1 **(C)**, 2 **(D)**, and 4 h **(E)** after treatment with paclitaxel (1 μM). Scale bars: 20 μm.

To examine the effects of paclitaxel and tempol on mitochondrial superoxide levels, the L1-6 DRGs were removed from a normal adult rat and DRG cells were cultured with the MitoSOX dye in a CO_2_ incubator. The MitoSOX reagent produces red fluorescence by mitochondrial superoxide but not by other free radicals. Paclitaxel increased the MitoSOX-detected red fluorescence intensity (**Figures 5A–E**). The 1 μM of paclitaxel significantly increased the fluorescence intensity at 2 (126%) and 4 h (132%) compared with before the treatment with paclitaxel (**Figures 5A,D,E**). These data indicate that paclitaxel increased the mitochondrial superoxide levels in the primary DRG cell culture. The DRG cells were alive at levels of 85–95% when treated with 1 μM of paclitaxel, but treatment with 5 μM of paclitaxel decreased cell viability to 62%. Therefore, we chose 1 μM of paclitaxel and 2 h for incubation time.

**FIGURE 6:**
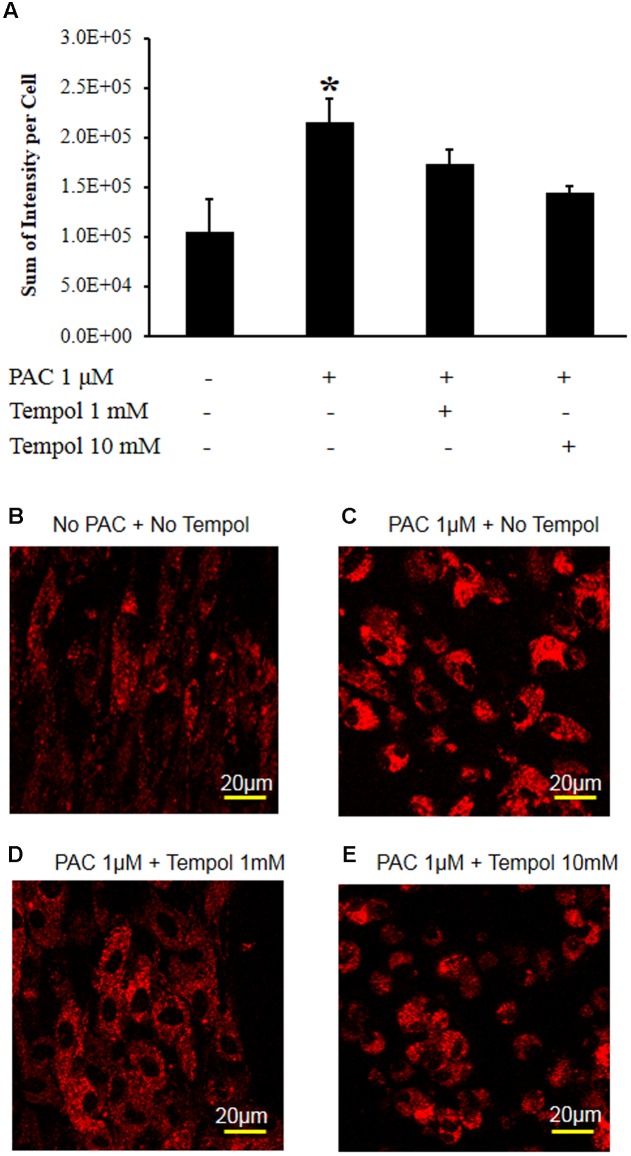
**Tempol decreased paclitaxel-induced mitochondrial superoxide levels in the primary DRG cell culture**. **(A)** The DRG cells were cultured in a chambered cover glass in DMEM media for live cell imaging. Paclitaxel (1 μM) significantly increased the MitoSOX-detected red fluorescence intensity in the primary DRG cell cultures at 2 h. Tempol (1 and 10 mM) decreased paclitaxel-induced red fluorescence intensity. The data are expressed as means ± standard deviations for three independent experiments. The asterisks indicate values that are significantly different (*P* < 0.05) from the values before treatment with paclitaxel as determined by the unpaired *t*-test. **(B)** The MitoSOX-detected red fluorescence without paclitaxel at 2 h. **(C)** The MitoSOX-detected red fluorescence at 2 h after treatment with paclitaxel (1 μM). **(D)** The MitoSOX-detected red fluorescence of DRG cells at 2 h after treatment with paclitaxel (1 μM) and tempol (1 mM). **(E)** The MitoSOX-detected red fluorescence of DRG cells at 2 h after treatment with paclitaxel (1 μM) and tempol (10 mM). Scale bars: 20 μm.

**Figure 6** shows that tempol decreased the MitoSOX-detected red fluorescence intensity at concentrations of 1 and 10 mM at 2 h after paclitaxel treatment. These data show that tempol decreased the mitochondrial superoxide levels in the primary DRG cell culture.

## Discussion

This study investigated the therapeutic and preventive effect of tempol in rats with PINP. Tempol produced analgesia by inhibiting mitochondrial superoxide level, p-PKC, p-NF-κB, PDE4D, IL-1β, and MCP-1 in DRGs. In addition, tempol prevented the development of PINP when given during the development phase of mechanical hyperalgesia. Therefore, the results suggest that tempol has potential for the treatment and prevention of chemotherapy-induced neuropathic pain.

It is well reported that oxidative stress plays an important role in neurodegenerative diseases (Wagner et al., 1998). Free radicals (superoxide, hydroxyl radicals, hydrogen peroxide, and peroxynitrite) are derivatives of molecular oxygen and nitrogen (Lander, 1997) and are produced by both mitochondrial oxidative metabolisms and several enzymes such as phospholipase A2, cytochrome P450, monoamine oxidase, and tyrosine hydroxylase. In addition, free radicals are removed by antioxidant systems including superoxide dismutase, catalase, glutathione, and glutathione peroxidase. Pathological condition is involved in oxidative stress by the increased production of free radicals and/or decreased antioxidants level (Floyd, 1999; Floyd and Hensley, 2002).

Fidanboylu et al. (2011) reported that single intraperitoneal injection of 100 mg/kg of tempol had no analgesic effect at 1, 3, and 24 h after injection, but 250 mg/kg had analgesic effect on established paclitaxel-induced hypersensitivity at 1 h after injection. In addition, daily systemic injection of 100 mg/kg of tempol for 13 days did not affect the development of paclitaxel-induced hypersensitivity (Fidanboylu et al., 2011). We think daily systemic injection of 100 mg/kg tempol is too low a dose for scavenging free radicals because of the lack of analgesic effect. In our study, the 100 and 200 mg/kg doses of tempol significantly increased the mechanical threshold for only 2 h. So the prolonged effect of tempol was induced by intraperitoneal injection at 200 mg/kg as an initial dose on 2 days and an intraperitoneal infusion at a rate of 10 mg/day as a maintaining dose because of the short analgesic effect of single systemic injection. This infusion dose produced analgesic effect without sedation.

We used a PINP model in rats because paclitaxel is a first-line drug for breast cancer and damages the peripheral nervous system, including nerve endings, peripheral nerves, and DRG (Dougherty et al., 2004). Paclitaxel cannot penetrate the blood-brain barrier and thus does not accumulate located in the central nervous system (Cavaletti et al., 2000). However, this drug does accumulate in DRGs (Cavaletti et al., 2000). The drug may cause damage to the sciatic nerve, nerve endings, and DRGs during the development of pain behavior (Peters et al., 2007b). Paclitaxel induces several events in the DRGs including (1) accumulation of immune cells such as macrophage and polymorphonuclear cells; (2) an increase in calcium channel subunits in the DRG (Matsumoto et al., 2006; Peters et al., 2007a; Xiao et al., 2007); (3) an increase in the expression of phospholipase A2, chemokine ligand 21b, complement components 1 and 3, and matrix metalloproteinase 3 (Nishida et al., 2008); and (4) an increase of inflammatory cytokines such as TNF-α, IL-1, and IL-6 (Ledeboer et al., 2007). These events may be responsible for changes in pain behavior.

In our study, paclitaxel increased the mitochondrial superoxide anion level in DRG cells, and tempol subsequently decreased the level. Superoxide anion is produced by the electron transport system in the mitochondria and is decreased by superoxide dismutase (Liu et al., 1996). Mitochondrial superoxide is generated as a by-product of electron transport chain in mitochondria. MitoSOX is a cationic derivative of dihydroethidium and is reactive with superoxide to produce 2-hydroxyethidium that induces a fluorescence (Zielonka et al., 2008). In our study, paclitaxel increased the superoxide anion level in the mitochondria and then induced oxidative stress in DRGs, which may explain the pain behavior. Superoxide anion forms hydrogen peroxide in the presence of tempol, which is similar to native superoxide dismutase (Reddan et al., 1993). Further, the nitroxide structure of tempol can facilitate metabolism of a wide range of free radicals and reactive nitrogen species. Indeed, tempol can protect cells from superoxide anion, and hydrogen peroxide (Reddan et al., 1992). Therefore, tempol improved pain behavior through scavenging free radicals in the DRGs.

Furthermore, paclitaxel increased the levels of p-PKC, p-NF-κB, PDE4D, IL-1β, and MCP-1 in the DRGs (**Figures 4A–F**). PDE4D is a subgroup of PDE4 that degrades the phosphodiesterase bond of cAMP and terminates the action of cAMP (Houslay and Adams, 2003). The activation of PDE4 produces inflammatory cytokines (IL-1β) and chemokines (MCP-1) in the immune cells (Boswell-Smith et al., 2006; Kobayashi et al., 2007; Kubo et al., 2012). Recently, rolipram, a selective PDE4 inhibitor, was shown to decrease pain behavior in rat PINP models (Kim et al., 2015). Also, paclitaxel has lipopolysaccharide-like action and then accumulates immune cells in the DRGs (Manthey et al., 1992) and increases intracellular calcium levels, which promote the change from PKC to p-PKC (Peters et al., 2007b; Xiao et al., 2007). The increase in activated PKC in the DRGs was observed after treatment with paclitaxel in a mouse model of PINP (He and Wang, 2015). In addition, p-NF-κB, an activated form of NF-κB, is translocated into the nucleus and then produced various proteins including inflammatory cytokines and chemokines such as TNF-α, IL-1β, and MCP-1, thereby inducing pain behaviors (Deshpande et al., 1997). In addition, paclitaxel was reported to increase the level of TNF-α in the DRG in rats (Kim et al., 2016). In this study, tempol reduced pain behaviors by decreasing the levels of p-PKC, p-NF-κB, PDE4D, IL-1β, and MCP-1 in the DRGs (but not those of TNF-α: data not shown).

Most importantly, tempol completely prevented the development of pain behaviors when it was administered on days 6–13 after the first injection of paclitaxel but not when it was administered on days 0–6. We found that tempol decreased oxidative stress, p-PKC, p-NF-κB, PDE4D, IL-1β, and MCP-1 in the DRGs. Therefore, induction of inflammatory cytokines/chemokines and oxidative stress may have been involved in the development of pain behaviors during days 6–13 after the first injection of paclitaxel. We plan to further investigate the temporal significance in studying the pathological mechanisms of the development of chemotherapy-induced neuropathic pain.

## Conclusion

Our study showed that systemic administration of tempol ameliorated and prevented neuropathic pain behavior in a rat model of PINP by inhibiting free radicals, p-PKC, p-NF-κB, PDE4D, IL-1β, and MCP-1 in the DRGs without inducing sedation. We conclude that oxidative stress and inflammatory processes are involved in both development and maintenance of chemotherapy-induced neuropathic pain.

## Author Contributions

HK contributed to conception, the design, data acquisition, analysis, interpretation, writing, and revising the manuscript. S-HH contributed to the design, data acquisition, analysis, and interpretation. SA contributed to conception, the design, data acquisition, analysis, interpretation, writing, and revising the manuscript.

## Conflict of Interest Statement

The authors declare that the research was conducted in the absence of any commercial or financial relationships that could be construed as a potential conflict of interest. The reviewer DP and handling Editor declared their shared affiliation, and the handling Editor states that the process nevertheless met the standards of a fair and objective review.
